# “Mixed Infection”—Report of Cases

**Published:** 1894-12

**Authors:** M. B. Hutchins

**Affiliations:** Clinical Lecturer on Skin Diseases and Syphilis, Atlanta Medical College, Atlanta Ga.


### ATLANTA
### MEDICAL AND SURGICAL JOURNAL.

VOL. XI. DECEMBER, 1894. No. 10.

EDITED BY
LUTHER B. GRANDY, M.D., AND MILLER B. HUTCHINS, M.D.,
WITH THE CO-OPERATION OF
H. V. M. MILLER, M.D., LL.D., VIRGIL O. HARDON, M.D.,
FLOYD W. McRAE, M.D., DUNBAR ROY, M.D.,
AND H. R. SLACK, M.D., PH.M.

### *ORIGINAL COMMUNICATIONS.*

### "MIXED INFECTION"—REPORT OF CASES.*

By M. B. HUTCHINS, M.D.,

Clinical Lecturer on Skin Diseases and Syphilis, Atlanta Medical College, Atlanta, Ga.

The term "mixed infection," as used here, applies to the combined infection with the virus of chancroid and with that of syphilis. This may occur simultaneously or successively; at the same time or at periods so near together as to cause the occurrence of a "mixed sore," or one lesion may so closely follow the other as, at any rate, to confuse the diagnosis. If the infection takes place simultaneously, the chancroid, having a shorter period of incubation, occurs first, and may heal before the appearance of the chancre, or persisting, aid to form the "mixed sore." If the chancre inoculation occurs first, the sores may partake of the characters of each. Finally, the chancroidal virus may be received in the chancre, or *vice versa*, thus also forming a "mixed sore." There is an element of uncertainty

*Read before the Tri-State Medical Association, Atlanta, Ga., October, 1894.

in the history of many venereal sores, and it is best to try to get along without depending upon the history, making your diagnosis clinically. I report two cases here which will illustrate some of the points of "mixed infection."

CASE 1.—Mulatto, male, aged twenty-one; first seen when there were nearly healed chancroids behind the corona glandis, and a walnut sized, fluctuating bubo in the right groin. The bubo was opened and appropriately treated. No especial attention was given the chancroids, as they appeared almost healed under aristol, but later calomel was applied. Within three weeks the greater part of the edge of the prepuce became studded with fine, sharply cut ulcers, the one uppermost being rather cartilaginous in feel. He become a patient at my clinic about this time, where, unfortunately, the record is not very complete. However, the hardness increased, and general glandular involvement occurred, together with the papular eruption and some throat trouble. Treatment was not closely followed, and about six months after the appearance of the chancre a marble sized, semi-elastic gumma developed beneath the skin of the dorsum of the penis. When seen a week later this was declining under the mixed treatment with bichloride of mercury and iodide of potassium and it soon disappeared.

CASE 2.—White, male, aged thirty. Referred to me by a Savannah physician, who had cauterized the lesions four days before. Five weeks before there was a slight laceration in the coronal sulcus on the right side during coitus. At the end of two weeks he noticed an excoriation there with the formation of pus. This lesion gradually enlarged to the size at the time when this record was made. About ten days after the appearance of the first lesion, a white, blister-like one occurred on the left side of the sulcus. At the end of two weeks the left inguinal glands were found enlarged. When I first saw the lesions they were just alike (this was four days after cauterization). each about the size of a dime, shallow, raw, round clean, their base rather hard.

There were three enlarged glands in the left inguinal region, none in the right, corresponding to the sore which appeared first. The case appears to have been one of chancroids at first, one following the other, but finally they gave evidence of syphilitic infection.

The last sore healed a little in advance of the first. Some basal hardness remained after healing. A little over five weeks after the appearance of the first, and about four weeks after the second, there was an increase in the number of enlarged glands in the left inguinal region, and the appearance of others in Scarpa's triangle. Two or three were also now perceptible in the right inguinal, one in the post-cervical region, and several about the ears. A scattering macular eruption on the abdomen and hips, a complete macular eruption on the back, a papule here and there on the forearms and back, a firm, scaly, livid-red papule upon the right eye-lid, one on the right and two or three on the left side of the neck, and one between the left pillars of the pharynx, now present. Slight general symptoms also present at times. The lesions on the right hip were perceptible some time before the occurrence of the general eruption, but were not sufficient to enable a positive diagnosis to be made.

It is hard to say whether the lesions in these two cases were due to simultaneous or successive infections, but the one appeared during the life of the other, typifying "mixed infection."

A venereal sore should not be cauterized while its nature is uncertain. Whether it should be cauterized at all depends upon the physician's judgment of the particular sore and its requirements. Anti-syphilitic treatment had best not be begun until the eruption appears, as its premature use may postpone or prevent an eruption, which is often the only sure guide to a diagnosis. If the lesion could be diagnosed positively at once, treatment might be begun at once, but it is seriously to be doubted if anything would be gained thereby with regard to the future of the case.

330 *Equitable Building.*

---

### APPLICATION FOR TOOTHACHE, NEURALGIA, RHEUMATISM, ETC.

The following (*Coll. and Clin. Record*) is an excellent application for local pain, as of toothache, neuralgia, rheumatism, etc.:

℞ Menthol,
Chloral,
Camphor, } aa .................................................grs. x.

Mix until liquefaction occurs.

Sig.—Apply locally.

